# Autism-epilepsy phenotype with macrocephaly suggests *PTEN*, but not *GLIALCAM*, genetic screening

**DOI:** 10.1186/1471-2350-15-26

**Published:** 2014-02-27

**Authors:** Maria Marchese, Valerio Conti, Giulia Valvo, Francesca Moro, Filippo Muratori, Raffaella Tancredi, Filippo M Santorelli, Renzo Guerrini, Federico Sicca

**Affiliations:** 1 Molecular Medicine Unit, IRCCS Stella Maris Foundation, Viale del Tirreno 331, Pisa, Calambrone 56128, Italy; 2 Child Neurology Unit, A. Meyer Pediatric Hospital, Viale Pieraccini 24, Florence 50139, Italy; 3 Clinical Neurophysiology Laboratory, IRCCS Stella Maris Foundation, Viale del Tirreno 331, Pisa, Calambrone 56128, Italy; 4 Developmental Psychiatry Unit, IRCCS Stella Maris Foundation, Viale del Tirreno 331, Pisa, Calambrone 56128, Italy

**Keywords:** Autism spectrum disorders, Autism-epilepsy phenotype, Macrocephaly, *GLIALCAM*, *PTEN*

## Abstract

**Background:**

With a complex and extremely high clinical and genetic heterogeneity, autism spectrum disorders (ASD) are better dissected if one takes into account specific endophenotypes. Comorbidity of ASD with epilepsy (or paroxysmal EEG) has long been described and seems to have strong genetic background. Macrocephaly also represents a well-known endophenotype in subgroups of ASD individuals, which suggests pathogenic mechanisms accelerating brain growth in early development and predisposing to the disorder. We attempted to estimate the association of gene variants with neurodevelopmental disorders in patients with autism-epilepsy phenotype (AEP) and cranial overgrowth, analyzing two genes previously reported to be associated with autism and macrocephaly.

**Methods:**

We analyzed the coding sequences and exon-intron boundaries of *GLIALCAM*, encoding an IgG-like cell adhesion protein, in 81 individuals with Autism Spectrum Disorders, either with or without comorbid epilepsy, paroxysmal EEG and/or macrocephaly, and the *PTEN* gene in the subsample with macrocephaly.

**Results:**

Among 81 individuals with ASD, 31 had concurrent macrocephaly. Head circumference, moreover, was over the 99.7th percentile (“extreme” macrocephaly) in 6/31 (19%) patients. Whilst we detected in *GLIALCAM* several single nucleotide variants without clear pathogenic effects, we found a novel *PTEN* heterozygous frameshift mutation in one case with “extreme” macrocephaly, autism, intellectual disability and seizures.

**Conclusions:**

We did not find a clear association between *GLIALCAM* mutations and AEP-macrocephaly comorbidity. The identification of a novel frameshift variant of *PTEN* in a patient with “extreme” macrocephaly, autism, intellectual disability and seizures, confirms this gene as a major candidate in the ASD-macrocephaly endophenotype. The concurrence of epilepsy in the same patient also suggests that PTEN, and the downstream signaling pathway, might deserve to be investigated in autism-epilepsy comorbidity. Working on clinical endophenotypes might be of help to address genetic studies and establish actual causative correlations in autism-epilepsy.

## Background

Autism spectrum disorders (ASD) are complex clinical conditions characterized by impairment in social and communicative functioning, and by restricted, repetitive and stereotyped behaviors [1]. Autism is often associated with other neurological conditions, particularly with seizures or paroxysmal EEG, defining a specific condition termed “Autism-Epilepsy Phenotype” (AEP) [2]. Although largely multifactorial in their background, both conditions are highly heritable and seem to have strong genetic underpinnings [3-6]. The recent development of next-generation sequencing techniques has heavily fostered the chance to identify genes with a putative causal role [3]. However, the polygenic/multifactorial nature of both ASD and epilepsy, and the heterogeneity of their phenotypes, which possibly underlie different etiologies, hinder the ability to demonstrate actual causalities and easy genotype-phenotype correlations. Within this complex framework, an attempt at identifying clinical endophenotypes could be of help to define diverse pathogenic subgroups pinpointing different genetic etiologies. A macrocephalic endophenotype has long been described, although the significance of this feature is still far from clear [7-10]. Recent findings indicate that cranial overgrowth in ASD appears in the first year of life suggesting possible mechanisms accelerating brain growth in early development and predisposing to autism [11-15]. We recently demonstrated that macrocephaly associated with global somatic overgrowth may predispose to seizures in a sample of two-hundred and six individuals with idiopathic ASD [16]. Also, cases of extreme macrocephaly in ASD have been correlated to mutations in the gene phosphatase and tensin homolog (*PTEN*) [17,18], making its testing the standard genetic screening for patients who present with autism and macrocephaly [19,20], although mutations account for only a small subset of the cases and have not been clearly correlated with susceptibility to seizures in ASD [21].

Mutations in the *GLIALCAM* gene (*HEPACAM*) have been recently identified in a spectrum of neurological conditions associated with macrocephaly. Biallelic, autosomal recessively inherited mutations in *GLIALCAM* were described in a proportion of the patients with the classic phenotype of Megalencephalic leukoencephalopathy with subcortical cysts (MLC2A, MIM 613925) associated with ataxia, spasticity, and intellectual disability [22] and no identifiable variants in the more commonly involved *MLC1* gene (MIM 605908). Interestingly, heterozygous, often *de novo*, mutations in *GLIALCAM* cause MLC2B (MIM 613926), a dominantly inherited condition characterized by onset of macrocephaly within the first year of life and mildly delayed motor development associated with white matter abnormalities on brain MRI [23-25]. In MLC2B the phenotype improves after the first year of life and head circumference in a few children normalizes. Most patients had delayed early motor and language development, which subsequently normalized in most, although some patients had mild residual hypotonia or clumsiness later in childhood. Intelligence was more variable, with intellectual disability in about half of the children, also associated with autism in a proportion of the cases. Seizures were a comorbid feature, though usually controlled well with medication. Brain MRI features were initially similar to those seen in MLC2A. On follow-up, however, all patients showed a significant improvement in the MRI changes, with loss of white matter swelling, disappearance of cysts in some cases, and lack of involvement of other brain regions.

The association of megalencephaly with seizures and autism spectrum disorders in a subset of MLC2B patients raised our interests on the role played by *GLIALCAM* in the ASD-macrocephaly-epilepsy endophenotype and prompted search for allelic variants in a subset of ASD patients, either with or without macrocephaly or seizures.

## Methods

### Patients

The sample was selected from our research database that included all ASD children who underwent EEG recordings between January 2010 and January 2013 in our clinical unit, and whose parents gave informed consent for collecting and storing clinical data and DNA samples for genetic analyses (Table 1). We selected from our database all patients with AEP and comorbid macrocephaly (n = 25), and a control sample of 25 consecutive AEP individuals with normal head circumference (HC). In addition, we have selected 31 consecutive ASD “simplex” (without seizures and with normal EEG), six of them (19%) with associated macrocephaly, in order to assess the relative frequency of gene variants in ASD in the absence of overt susceptibility to epilepsy. The diagnosis of ASD was performed in all patients according to the Diagnostic and Statistical Manual of Mental Disorders, Fourth Edition, Text Revision (DSM-IV TR) criteria for Pervasive Developmental Disorders, and confirmed, whenever possible, with the Autism Diagnostic Observation Schedule-Generic (ADOS-G). History of seizures and video-EEG recordings during awake and sleep were evaluated by two independent clinicians. HC was obtained in all children by placing a tape measure over the maximum occipital-frontal circumference, and was plotted for reference on standard growth charts [26]. Macrocephaly was defined when HC was over the 97th percentile, and labeled as “extreme” when over the 99.7th percentile [21], corresponding to +3 standard deviations (2010 Centers for Disease Control and Prevention growth charts) [27]. Karyotype and Fragile-X testing were performed in 76/81 (93.8%) and 77/81 (95.1%) patients, respectively and were normal in all. CGH-array screening, performed in 15/81 (18.5%) cases, was also negative.

**Table 1:** Clinical sample

	**AEP with macrocephaly**	**AEP without macrocephaly**	**ASD “simplex” with macrocephaly**	**ASD “simplex” without macrocephaly**
**Sample size**	25	25	6	25
**Gender**				
- M	20 (80%)	21 (84%)	4 (66.7%)	24 (96%)
- F	5 (20%)	4 (16%)	2 (33.3%)	1 (4%)
**Age (years)**				
- range	2.9-20.8	4.0-15.7	3.0–9.9	2.4–10.0
– mean; SD	9.7; 5.2	7.6; 3.0	5.3; 2.4	5.2; 1.8
**AEP subtype:**				
– Seizures	13 (52%)	13 (52%)	–	–
– EEG abnormalities without seizures	12 (48%)	12 (48%)	–	–
**Age at seizure onset**				
–range	3.0–17.9	0.4–15	–	–
–mean; SD	9.2; 4.9	4.5; 4.4	–	–
**Type of seizures**				
–Focal	10/13 (76.9%)	7/13 (53.8%)	–	–
–Generalized	3/13 (23.1%)	4/13 (30.8%)	–	–
–Spasms	–	2/13 (15.4%)	–	–
**Type of EEG abnormalities**				
–Focal	15/22 (68.2%)	14/23 (60.9%)	–	–
–Multifocal	6/22 (27.3%)	9/23 (39.1%)	–	–
–Diffuse	1/22 (4.5%)	–	–	–

### Genetic analyses

Total genomic DNA from peripheral blood was obtained from patients using standard purification protocols. The coding exons and exon-intron boundaries of *GLIALCAM* (accession number NM_152722.4) were PCR amplified using oligonucleotide primers and conditions outlined elsewhere [22]. The PCR products were purified with ExoSap (USB, Cleveland, OH) and bidirectionally sequenced using the BigDye v3.1 chemistry (Applied Biosystems Foster City, CA). Chromatograms were analyzed using SeqScape Software (Life Technologies). Synonymous, missense and splice site variations were systematically evaluated for modifications of exonic splicing enhancers (Polyphen analysis, http://genetics.bwh.harvard.edu/pph/; SIFT analysis (http://sift.jcvi.org/); Mutation Taster analysis, http://www.mutationtaster.org/; ESEfinder, http://rulai.cshl.edu/cgi-bin/tools/ESE3/esefinder.cgi) or consensus splicing sequences in order to determine the splice site score (http://rulai.cshl.edu/new_alt_exon_db2/HTML/score.html and http://www.fruitfly.org/seq_tools/splice.html). We also analyzed the exons and the flanking intronic regions corresponding to the *PTEN* gene (NM_000314.4) in the 31 individuals with macrocephaly. PCR reactions were performed using 50 ng of genomic DNA as template and FastStart Taq DNA Polymerase (Roche, Mannheim, Germany). PCR products were purified and bidirectionally sequenced as stated above. Primers used for *PTEN* mutation screening are available upon request.

### Ethics statement

This study was approved by the Research Ethics Committee of the IRCCS Fondazione Stella Maris, Pisa (Italy) in compliance with the Helsinki Declaration and local legislation.

## Results

We tested for *GLIALCAM* variants 81 individuals with ASD (50 AEP and 31 ASD “simplex”), 31 of them with concurrent macrocephaly (see Table 1 for more clinical details). Among macrocephalic individuals, 6/31 (19%) were labeled as “extreme”. Whilst we did not identify any nucleotide variant with presumable pathogenic effects, we detected several single nucleotide variants (SNV) already reported in polymorphic databases. In particular, we found two non-synonymous variations, already listed in dbSNP (http://www.ncbi.nlm.nih.gov/SNP/), namely, p.M218V (*rs10790715*) and p.N324S (*rs116102273*). In our group of patients, the V218 allele occurred in 75% of the cases, whereas S324 was present in 3.10% of the alleles, without significant correlation with phenotypic characteristics (such as presence of macrocephaly and/or seizures/EEG abnormalities), and in line with figures reported in the Exome Variant Server polymorphic database (http://evs.gs.washington.edu/EVS/). Both changes had a likely benign effect upon *in silico* prediction analyses. In fact p.M218V result to be benign because the SIFT score was 1, Polyphen showed a score of 0.00. Similar results were seen for p.N324S with Polyphen and SIFT scores of 0.00 and 0.38, respectively. Additional analyses in MutationTaster indicated that both variants behave as benign polymorphisms.

**Figure 1:**
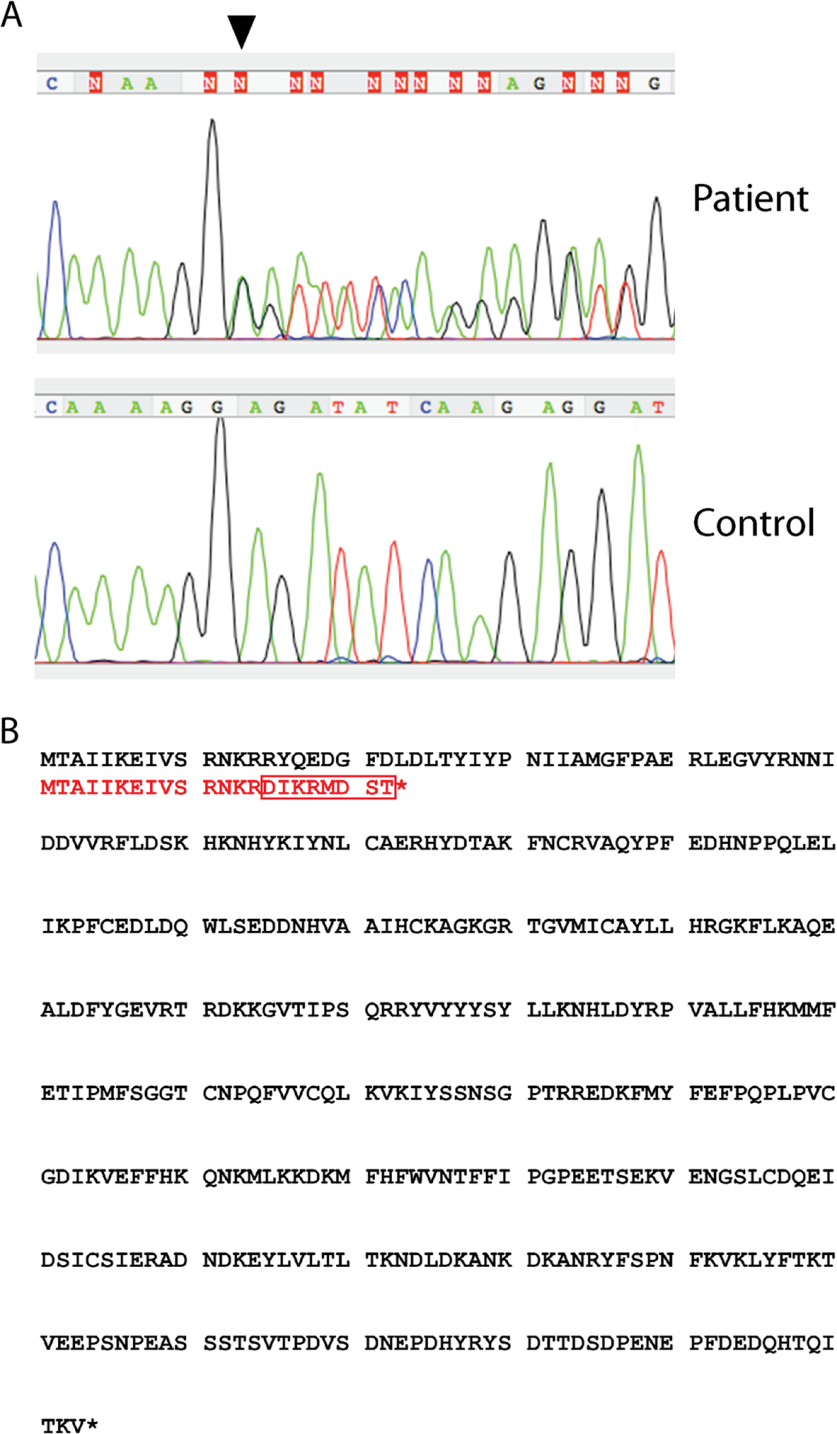
DNA sequence electropherograms showing the novel frameshift mutation in PTEN (c.43delA, black arrowhead in A), and blast alignment of the wild-type PTEN protein (in black) and the mutant protein (in red) (B).

Genetic screening of *PTEN* in the whole sample of ASD-macrocephaly, either with or without epilepsy\EEG abnormalities, revealed the presence of a novel heterozygous frameshift mutation (c.43delA; p.R15Dfs*9) (Figure 1A) in one case with “extreme” macrocephaly, autism, intellectual disability and history of a first unprovoked seizure. This boy was born at term, from non-consanguineous parents, after uneventful pregnancy and delivery. He experienced, since the first year, poor social interaction and communication. Psychomotor development was delayed and he walked without support at 30 months. At the age of 5 years and 9 months, he exhibited a first generalized tonic-clonic seizure and underwent valproic acid (VPA) treatment. When he came to our attention, at the age of 6 years and 7 months, clinical evaluation showed diffuse hypotonia, absence of speech, moderate intellectual disability, severe behavior problems (frustration intolerance, aggressive behaviors), stereotypies and severe disorder of social interaction consistent with DSM-IV-TR criteria for ASD. Head circumference was 4.24 SD above the mean (> 99.7th percentile, “extreme” macrocephaly). He was still under VPA therapy, but he had not experienced other seizures. Wake electroencephalogram, brain MRI, karyotype, FRAXA and FRAXE analyses were normal.

The *PTEN* mutation detected in this boy determined the replacement of eight amino acids (aa 15–22) with respect to the canonical protein sequence and predicted the introduction of an early stop codon with a severely truncated protein (Figure 1B). The mutation was not reported in the Kaviar2 database (http://db.systemsbiology.net/kaviar/cgi-pub/Kaviar2.pl). Since parents refused genetic testing, we could not determine whether the variant was inherited or de novo.

## Discussion

GlialCAM (glial cell adhesion molecule) is an Ig-like protein of still poorly characterized function, possibly involved in cell-matrix interactions and in cell motility [28]. It is predominantly expressed at cell-cell contacts between end-feet of astrocytes, and probably between oligodendrocytes and astrocytes [22]. Disease-causing mutations of *GLIALCAM* are associated with a phenotypic spectrum including macrocephaly, leukoencephalopathy, and epilepsy, with or without autism, as well as intellectual disability and psychomotor deterioration. GlialCAM forms homo- and hetero-complexes that are reduced by MLC-related mutations with ensuing defective trafficking to cell junctions [29]. GlialCAM has been recently identified as a Cl^−^ channel ClC-2 partner, targeting it to cell junctions and modulating its conductance [30]. CLC-2 is a hyperpolarization-activated and osmosensitive channel [31,32] possibly playing a role in glial-mediated fluid and ion homeostasis, and in the maintenance of low extracellular potassium during high neuronal activity [30,33-35]. Analogously to Kir4.1 defects, which predispose to autism-epilepsy comorbidity by altering the astrocytic-dependent potassium buffering [36], a defective ion trafficking due to GlialCAM mutations might cause osmotic imbalance and fluid accumulation [30,37,38] leading to the aforementioned macrocephalic-related spectrum of diseases. Taken together, these evidences led us to consider *GLIALCAM* as a candidate gene in Autism-epilepsy associated with macrocephaly, and to screen a sample of individuals with ASD either with or without this comorbid endophenotype. Our negative findings indicate a lack of clear association with autism, macrocephaly and seizure susceptibility, although they cannot completely rule out the involvement of *GLIALCAM* in this endophenotype due to the relatively small sample size. We cannot exclude, moreover, that GlialCAM may participate in a more complex glial protein network, contributing to dysregulation of astrocyte homeostasis and cell growth in AEP.

Finally, we also found a novel *PTEN* frameshift mutation in one patient with AEP associated with extreme macrocephaly. Mutations in *PTEN* have already been associated with the development of many cancers as well as non-tumor phenotypes, including macrocephaly and ASD [19,39,40]. *PTEN* is an important negative regulator of PI3K/AKT/mTOR signaling pathway, which has roles in controlling cell growth, survival and proliferation [41,42]. Mutations in genes of this pathway have been associated with other conditions encompassing epilepsy and/or ASD, such as the Tuberous Sclerosis complex [43] and other megalencephaly syndromes [44,45], suggesting that disinhibited PTEN/PI3K/AKT/mTOR signaling may represent a key mechanism implicated in the pathogenesis of ASD, seizures and brain overgrowth. Mice with targeted inactivation of the *Pten* gene in differentiated neurons of the cerebral cortex and hippocampus also demonstrated macrocephaly, abnormal social interaction and exaggerated responses to sensory stimuli [46]. Ablating *Pten* broadly during developmental stages causes premature death in mice, often accompanied by severe epileptic activity [20]. Although we found a single patient harboring a *PTEN* mutation, this gene remains a likely candidate in AEP with extreme macrocephaly.

## Conclusions

We did not find a clear pathogenic link between *GLIALCAM* variants and AEP-macrocephaly comorbidity, suggesting that *GLIALCAM* genetic screening is currently not indicated in patients with ASD. However, the relatively small size of our sample prevents to draw definitive conclusions. Although next-generation sequencing techniques offer enormous opportunities to discover the genetic background of autism-related disorders, their extreme phenotypic heterogeneity and the difficulty in recruiting pathophysiologically homogeneous samples, make it difficult to establish actual causative correlations. A candidate-gene approach, although less performing, could potentially be applicable when justified by a clear clinical and/or pathophysiological rationale. Working on clinical endophenotypes might help - in both approaches - to define more valid genotype-phenotype correlations. One example is, in this work, the identification of a deleterious mutation of *PTEN* in a patient with “extreme” macrocephaly, in one out of 6 cases with this somatic endophenotype. The concurrence of epilepsy in the same patient also suggests that *PTEN* gene, and the downstream PI3K/AKT/mTOR pathway, deserves to be further investigated in autism-epilepsy comorbidity. Accurate definition of clinical endophenotypes might be an approach to untangle the complex genotype-phenotype correlations in AEP.

## Abbreviations

ASD: Autism spectrum disorders; EEG: Electroencephalogram; AEP: Autism-epilepsy phenotype; HC: Head circumference.

## Competing interests

The authors declare that they have no competing interests.

## Authors’ contributions

MM carried out the molecular genetic studies (*GLIALCAM*) and participated in drafting the manuscript. VC carried out the molecular genetic studies (*PTEN*) and participated in drafting the manuscript. GV was involved in the acquisition and analysis of clinical data and in drafting the manuscript. FrM contributed to carry out the molecular genetic studies (*GLIALCAM*) and analyze genetic data. RT was involved in the collection of patients and in the acquisition and analysis of clinical data. FiM and RG participated in the coordination of the study and revised the draft critically. FMS and FS conceived the study, participated in its design and coordination and contributed to draft the manuscript. All authors read and approved the final manuscript.

## Pre-publication history

The pre-publication history for this paper can be accessed here:

http://www.biomedcentral.com/1471-2350/15/26/prepub
